# Clinical Impact of Kidney Function on Presepsin Levels

**DOI:** 10.1371/journal.pone.0129159

**Published:** 2015-06-01

**Authors:** Takanobu Nagata, Yoshinari Yasuda, Masahiko Ando, Tomoko Abe, Takayuki Katsuno, Sawako Kato, Naotake Tsuboi, Seiichi Matsuo, Shoichi Maruyama

**Affiliations:** 1 Department of Nephrology, Nagoya University Graduate School of Medicine, Nagoya, Japan; 2 Center for Advanced Medicine and Clinical Research, Nagoya University Hospital, Nagoya, Japan; School of Public Health of University of São Paulo, BRAZIL

## Abstract

**Objective:**

Presepsin is highlighted as a diagnostic and prognostic marker of sepsis. Little information is available regarding the accurate association between presepsin levels and the degree of kidney function. We analyzed presepsin levels in patients with a glomerular filtration rate (GFR) in the categories G1 to G5, evaluated via inulin renal clearance test, and receiving hemodialysis (HD).

**Methods:**

Patients who were not receiving HD were included if they had undergone inulin renal clearance measurements for the accurate measurement of GFR (measured GFR), and patients who were receiving hemodialysis (HD) were included if they had anuria. Exclusion criteria were infection, cancer, liver disease, autoimmune disorders, or steroid or immunosuppressant use. GFR category was defined as follows; G1: GFR ≥ 90 ml/min/1.73m^2^, G2: GFR = 60 to 90 ml/min/1.73m^2^, G3: GFR = 30 to 60 ml/min/1.73m^2^, G4: GFR = 15 to 30 ml/min/1.73m^2^, G5: GFR ≤ 15 ml/min/1.73m^2^.

**Results:**

Seventy-one patients were included. The median (IQR) presepsin values of patients in each GFR category were as follows: G1 + G2: 69.8 (60.8–85.9) pg/ml; G3: 107.0 (68.7–150.0) pg/ml; G4: 171.0 (117.0–200.0) pg/ml; G5: 251.0 (213.0–297.5) pg/ml; and HD: 1160.0 (1070.0–1400.0) pg/ml. The log-transformed presepsin values, excluding patients receiving HD, inversely correlated with the measured GFR (Pearson’s correlation coefficient = -0.687, P < 0.001). The multivariate analysis revealed that measured GFR and hemoglobin levels significantly correlated with elevated presepsin levels.

**Conclusion:**

Presepsin levels were markedly high in patients receiving HD, similar to values seen in patients with severe sepsis or septic shock. In patients who were not receiving HD, presepsin levels increased as GFR decreased. Thus, the evaluation of presepsin levels in patients with chronic kidney disease requires further consideration, and a different cutoff value is needed for diagnosing sepsis in such patients.

## Introduction

Sepsis is a major cause of mortality in patients presenting to the emergency department (ED) or intensive care unit (ICU). Early recognition and treatment initiation are essential for improving the prognosis of patients with sepsis [1,2,3]. Various biomarkers such as procalcitonin (PCT) have been used for diagnosing sepsis [4,5]. Although PCT level often indicates the presence of infection, its levels are also elevated in various conditions that induce systemic inflammatory response syndrome, such as severe trauma, burn injury, or surgical procedures [6,7]. Therefore, it is necessary to develop a biomarker that is more specific and that can be used for the earlier detection of sepsis compared to PCT.

Presepsin is the soluble N-terminal fragment of the cluster of differentiation (CD) marker protein CD14, which is the receptor for lipopolysaccharide (LPS) and LPS-binding protein complexes [8]. Recently, presepsin was reported as valuable for the early diagnosis of sepsis and the evaluation of sepsis severity, and its levels remain unaffected by conditions such as trauma, burn injury, or surgical procedures [8,9]. The diagnostic cutoff levels for sepsis varied among different studies, but most reports suggest approximate levels of 400–600 pg/ml [8,10].

In addition, based on experiments with septic animal models, presepsin levels increase 2 hours after the onset of infection, which is earlier than the elevation of PCT levels [11,12].

Moreover, some studies have reported that the measurement of presepsin levels is useful for predicting the prognosis of septic patients [10,13,14,15]. Because of these characteristics, presepsin is being used in various clinical situations.

However, there is a concern that presepsin level is affected by kidney function. Presepsin is a 13 kDa protein. Although its exact in vivo activity is unclear, it is presumed from its molecular weight that presepsin is filtered by the glomerulus, reabsorbed, and catabolized within proximal tubular cells [16]. Theoretically, it has been proposed that presepsin levels increase as kidney function decreases. Chenevier-Gobeaux et al. [16] measured presepsin levels in patients who presented to the ED with mild illness without acute infection. They showed that presepsin levels were elevated in most patients with a decreased estimated glomerular filtration rate (eGFR; <60 ml/min/1.73m^2^). Behnes et al. [17] reported that, in an internal ICU, presepsin levels significantly correlated with serum creatinine levels and the number of days on renal replacement therapy, which are both related to kidney function. Nakamura et al. [18] retrospectively analyzed presepsin levels in patients with or without sepsis presenting in the ICU, and found that that presepsin levels were markedly high in patients with renal failure and end-stage kidney disease. Recently, Masson et al. [19] reported that higher serum creatinine was the strongest determinant of presepsin levels in ICU patients. Because these reports studied patients presenting to the ED or ICU, various factors could have affected presepsin levels. Additionally, these studies evaluated kidney function based on eGFR or urine output, and therefore, the accurate association between presepsin levels and GFRs was not elucidated. Considering this, we conducted a cross-sectional study to investigate the effect of GFR, measured precisely, and hemodialysis (HD) dependence on presepsin levels.

## Materials and Methods

### Patients

Study participants were outpatients who had visited the department of Nephrology at Nagoya University Hospital and Ogaki Municipal Hospital between 2009 and 2013. The study included patients who had undergone inulin renal clearance measurements for the accurate evaluation of GFR and patients with anuria receiving HD with a high-flux dialyzer. All patients receiving HD had dialysis with bicarbonate three times per week. Planned dialysis duration was 240 or 300 minutes, with a blood flow rate of 200 ml/min and a dialysate flow rate of 500 ml/min. Ultrapure dialysate (a bacterial count of < 0.1 colony-forming units /mL and less than 0.03 endotoxin units /ml) was used [20]. Gamma-ray sterilization was performed for all dialyzers and reuse was not permitted. Exclusion criteria were infection, cancer, liver disease, autoimmune disorder, and steroid or immunosuppressant use. The study participants were recruited consecutively from the list of participants who met the study criteria.

The study protocol and consent procedure were approved by the ethics committees of Nagoya University and Ogaki Municipal Hospital according to the Declaration of Helsinki. The approval number was 1135–15. Written informed consent was obtained from every participant.

### Data and sample collection

Patients’ clinical characteristics and data were collected retrospectively from their medical records. The eGFR was calculated using the equation generated by the Japanese Society of Nephrology: eGFR (ml/min/1.73 m^2^) = 194 × Scr^-1.094^ × Age^-0.287^ × 0.739 (if female) [21]. The inulin renal clearance (measured GFR) was measured using the simple method previously reported as accurate enough for measuring GFR in clinical practice [22,23].

GFR was categorized according to the KDIGO 2012 clinical practice guideline for the evaluation and management of chronic kidney disease [24]. GFR category was defined as follows; G1: GFR ≥ 90 ml/min/1.73m^2^, G2: GFR = 60 to 90 ml/min/1.73m^2^, G3: GFR = 30 to 60 ml/min/1.73m^2^, G4: GFR = 15 to 30 ml/min/1.73m^2^, G5: GFR ≤ 15 ml/min/1.73m^2^. High-sensitivity C-reactive protein (CRP) was measured by latex-enhanced immuno-nephelometric assay and the limit of detection level was 0.005 mg/dl. Procalcitonin values were analyzed in patients not receiving HD by electrochemiluminescence immunoassay and the limit of detection level was 0.02 ng/ml.

Plasma samples containing ethylenediaminetetraacetic acid were collected at the time of inulin renal clearance measurement, just before inulin infusion, in a fasting state. In patients receiving HD, plasma samples were collected immediately before an HD session and two days after the prior HD. Plasma samples were stored at -80°C until presepsin measurements were performed.

### Presepsin measurement

Plasma presepsin concentrations were measured using a compact automated immunoanalyzer (PATHFAST; LSI Medience Corporation, Tokyo, Japan) based on a chemiluminescent enzyme immunoassay [25,26]. The measurement range of the assay is 20–20,000 pg/ml.

### Statistical analysis

Normally distributed variables are expressed as means and standard deviations (SD) and were compared using one-way analysis of variance. Nonparametric variables were expressed as medians and interquartile ranges and compared using Kruskal-Wallis tests with Bonferroni *post hoc* tests. Categorical variables were expressed as percentages and were compared using Fisher’s exact test. Because the distribution of presepsin values was skewed, values from a logarithmic transformation were used in univariate and multivariate analyses. The univariate analysis for presepsin levels was conducted using Pearson’s or Spearman’s rank correlation coefficients. A multivariate linear regression model for presepsin levels was constructed using a backward stepwise method of selection. All P values were two-tailed, and P < 0.05 was considered statistically significant.

All statistical analyses were performed using EZR (Saitama Medical Centre, Jichi Medical University, Saitama, Japan; http://www.jichi.ac.jp/saitama-sct/SaitamaHP.files/statmedEN.html), which is a graphical user interface for R (The R Foundation for Statistical Computing, Vienna, Austria, version 2.13.0) [27].

## Results

**Fig 1:**
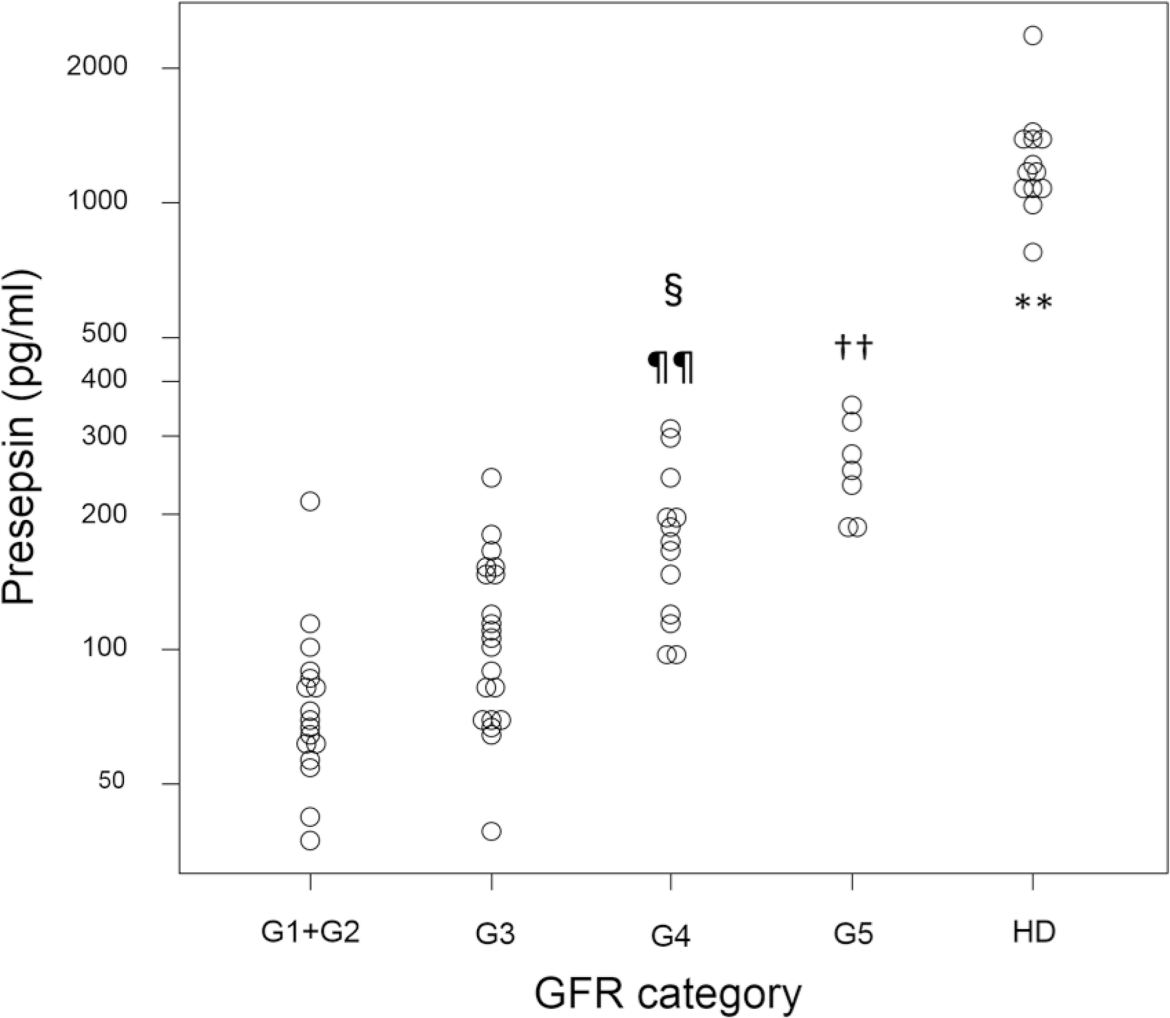
Dot plot of presepsin values of patients in the different GFR categories or of patients receiving HD. G1: GFR ≥ 90 ml/min/1.73m^2^, G2: GFR = 60 to 90 ml/min/1.73m^2^, G3: GFR = 30 to 60 ml/min/1.73m^2^, G4: GFR = 15 to 30 ml/min/1.73m^2^, G5: GFR ≤ 15 ml/min/1.73m^2^, HD: hemodialysis. **P <0.01 compared to any other GFR category. ††P <0.01 compared to G3 and G2+G1. ¶¶P <0.01 compared to G1+G2. §P <0.05 compared to G3.

**Fig 2:**
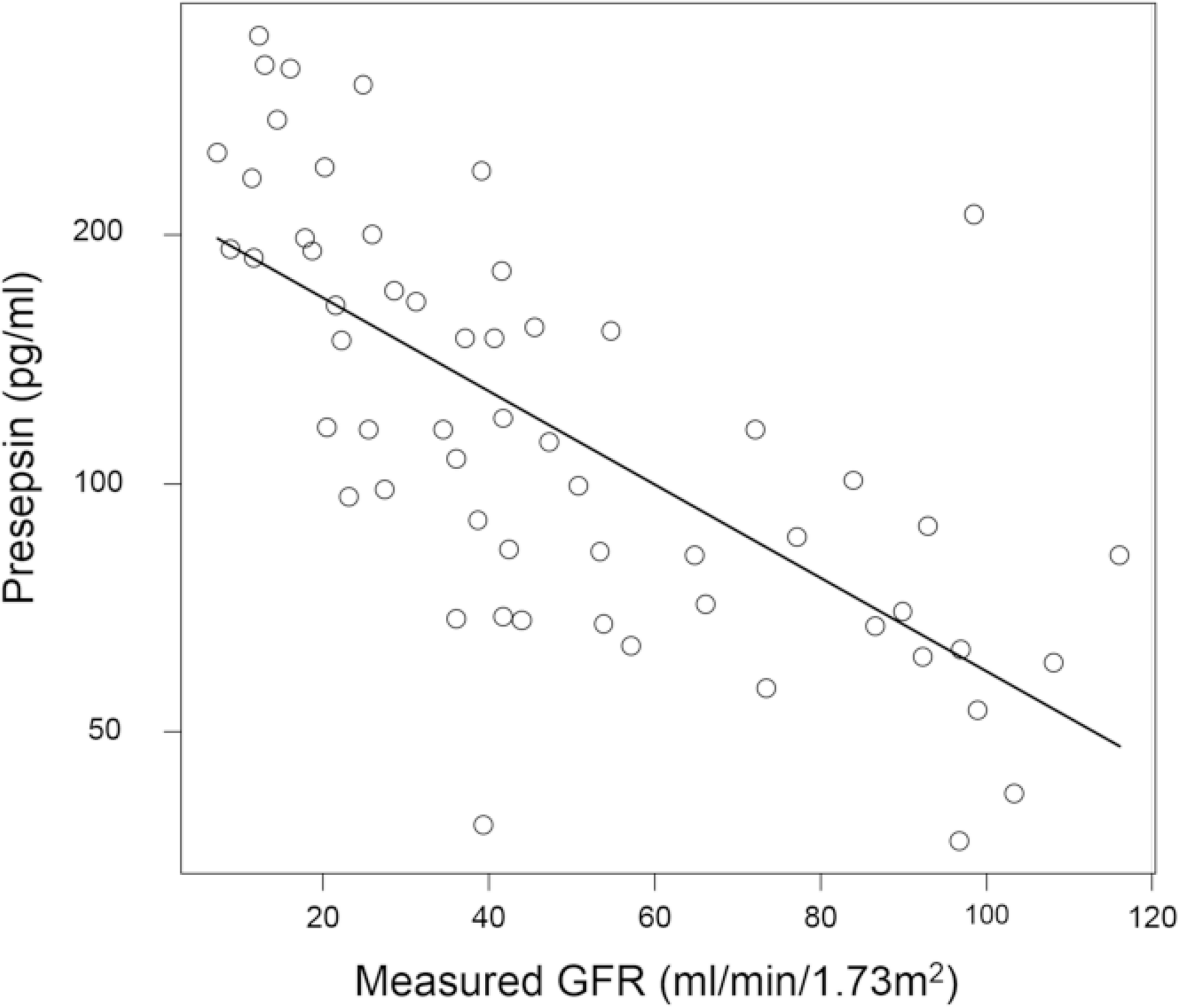
Correlation between the log-transformed presepsin values and measured GFR in patients not receiving hemodialysis. N = 58, Pearson’s correlation coefficient = -0.687, 95% CI = -0.803 to -0.521, P <0.001.

Clinical characteristics of the patient population and subgroups are presented in Table 1. There were 12 kidney transplantation donor candidates in the G1 + G2 group. Patients receiving HD had markedly high presepsin levels compared to those not receiving HD (Fig 1). The median and interquartile range of presepsin values were as follows: G1 + G2: 69.8 (60.8–85.9) pg/ml; G3: 107.0 (68.7–150.0) pg/ml; G4: 171.0 (117.0–200.0) pg/ml; G5: 251.0 (213.0–297.5) pg/ml; and HD: 1160.0 (1070.0–1400.0) pg/ml. Presepsin levels in patients in the G4 or G5 category were significantly higher than those in patients in the G1 + G2 and G3 categories. Presepsin levels were higher in patients in the G3 category than patients in the G1 + G2, and in the G5 than in the G4, but differences in these values were not statistically significant. The log-transformed presepsin values in patients not receiving HD correlated well with measured GFR (Pearson’s test = -0.687, P < 0.001) (Fig 2).

**Table 1:** Clinical characteristics of the study participants and comparison of patients in the different GFR categories or receiving HD.

		GFR category					
	Overall	G1+ G2	G3	G4	G5	HD	P value
Number of patients	71	17	21	13	7	13	
Age (years)	63.4 (11.4)	60.2 (10.3)	63.2 (11.9)	67.2 (8.7)	60.8 (16.3)	65.8 (11.7)	0.46
Gender (male/female)	42/29	7/10	16/5	11/2	4/3	4/9	0.012
Height (cm)	160.2 (8.0)	160.3 (7.7)	161.7 (6.6)	165.5 (6.9)	159.9 (6.1)	152.3 (7.5)	< 0.001
Weight (kg)	60.3 (11.4)	59.9 (11.3)	63.5 (8.3)	66.3 (11.0)	60.6 (14.3)	49.6 (8.2)	0.001
Diabetes mellitus, n (%)	20 (28.2)	4 (23.5)	8 (38.1)	4 (30.8)	1 (14.3)	3 (23.1)	0.776
Total protein (g/dl)	6.97 (0.53)	7.1 (0.4)	7.0 (0.5)	6.9 (0.6)	7.1 (0.3)	6.5 (0.3)	<0.009
Serum albumin (g/dl)	3.9 (0.3)	4.2 (0.2)	3.9 (0.3)	3.8 (0.4)	3.8 (0.2)	3.8 (0.3)	< 0.001
Aspartate aminotransferase (U/l)	20.6 (6.9)	22.2 (6.2)	21.5 (7.6)	20.2 (6.7)	20.8 (6.0)	17.4 (7.3)	0.421
Alanine aminotransferase (U/l)	16.6 (7.7)	18.1 (3.9)	18.8 (10.0)	15.5 (6.6)	17.5 (7.4)	11.8 (6.5)	0.101
Uric acid (mg/dl)	6.89 (1.61)	5.1 (1.4)	7.0 (0.8)	6.9 (0.9)	7.5 (1.5)	8.4 (1.3)	< 0.001
Urea nitrogen (mg/dl)	32.5 (19.8)	13.2 (3.7)	22.2 (5.8)	35.1 (11.1)	45.8 (12.7)	64.3 (10.0)	< 0.001
Serum creatinine (mg/dl)	3.12 (3.75)	0.68 (0.22)	1.15 (0.27)	2.09 (0.43)	3.20 (0.90)	10.47 (2.52)	< 0.001
estimated GFR (ml/min/1.73m^2^)	50.4 (29.1)	84.2 (23.9)	50.1 (12.4)	25.7 (5.4)	15.5 (2.6)	NA	< 0.001
measured GFR (ml/min/1.73m^2^)	48.2 (30.2)	89.3 (14.7)	43.2 (7.2)	22.5 (3.8)	11.3 (2.5)	NA	< 0.001
Glucose (mg/dl)	104 (26)	100 (25)	108 (31)	110 (32)	95 (12)	101 (15)	0.693
C reactive protein (mg/dl)	0.08 (0.07)	0.06 (0.05)	0.09 (0.07)	0.06 (0.05)	0.07 (0.08)	0.12 (0.11)	0.285
Procalcitonin (ng/ml)	0.05 (0.03)	0.04 (0.04)	0.05 (0.04)	0.07 (0.04)	0.08 (0.02)	NA	0.046
White blood cells (10^6^/l)	5850 (1632)	5329 (1546)	6319 (1675)	5823 (1146)	6342 (2310)	5537 (1636)	0.339
Hemoglobin (g/dl)	12.8 (1.6)	13.8 (1.3)	13.5 (1.6)	12.0 (1.4)	11.0 (1.4)	12.1 (1.2)	< 0.001
Platelets (10^10^/l)	20.4 (5.6)	21.6 (4.3)	20.1 (4.5)	20.8 (5.8)	22.9 (6.4)	17.4 (7.5)	0.212

NA: data not available. Continuous variables are expressed as mean (standard deviation). P values were calculated using one-way analysis of variance or Fisher’s exact test. G1: GFR ≥ 90 ml/min/1.73m^2^, G2: GFR = 60 to 90 ml/min/1.73m^2^, G3: GFR = 30 to 60 ml/min/1.73m^2^, G4: GFR = 15 to 30 ml/min/1.73m^2^, G5: GFR ≤ 15 ml/min/1.73m^2^, HD: hemodialysis.

Univariate analysis found that levels of serum albumin, serum uric acid, serum urea nitrogen, serum creatinine, and hemoglobin, as well as eGFR and measured GFR, significantly correlated with presepsin levels. We excluded levels of serum creatinine, serum urea nitrogen, and eGFR from the multivariate model because the correlation coefficients between those variables and measured GFR were high (0.778, 0.921, and -0.771, respectively; Pearson’s test). Weak correlation was found between measured GFR and serum albumin, uric acid and hemoglobin levels (correlation coefficients were 0.531, 0.663 and 0.437, respectively). In this study, we added all variables with correlation coefficients < 0.7 to the multivariate model. We found that measured GFR and hemoglobin levels were significantly associated with elevated presepsin levels (Table 2).

**Table 2:** Univariate analysis and multivariate linear regression analysis of the increase in log-transformed presepsin values.

	Univariate		Multivariate		
Candidate variables	Correlation coefficient	P value	Beta regression coefficient	Standard error	P value
Age	0.087	0.513^a^			
Gender (female = 1)	-0.110	0.404^b^			
Height (cm)	0.054	0.683^a^			
Weight (kg)	0.094	0.481^a^			
Diabetes mellitus (yes = 1)	0.183	0.168^b^			
Total protein (g/dl)	-0.065	0.624^a^			
Serum albumin (g/dl)	-0.370	0.004^a^			
Aspartate aminotransferase (U/l)	0.019	0.885^a^			
Alanine aminotransferase (U/l)	-0.033	0.803^a^			
Uric acid (mg/dl)	0.505	<0.001^a^			
Urea nitrogen (mg/dl)	0.715	<0.001^a^			
Serum creatinine (mg/dl)	0.722	<0.001^a^			
estimated GFR (ml/min/1.73m^2^)	-0.638	<0.001^a^			
measured GFR (ml/min/1.73m^2^)	-0.687	<0.001^a^	-0.00483	0.00085	<0.001
Glucose (mg/dl)	0.191	0.151^a^			
C-reactive protein (mg/dl)	0.005	0.968^a^			
Procalcitonin (ng/ml)	0.383	0.003^a^			
White blood cell count (10^6^/l)	0.117	0.381^a^			
Hemoglobin (g/dl)	-0.482	<0.001^a^	-0.032	0.014	0.035
Platelet count (10^10^/l)	0.004	0.973^a^			

a: Pearson’s correlation coefficients

b: Spearman’s correlation coefficients

After obtaining these results in patients receiving HD, we additionally measured presepsin levels in the same patients on another day, immediately before and after HD, two days after the prior HD. Presepsin levels decreased significantly from 1510 (1280–1670) pg/ml before HD to 753 (542–1210) pg/ml after HD (P < 0.001).

## Discussion

This study evaluated the clinical impact of kidney function on presepsin levels and found that presepsin level inversely correlated with GFR. In particular, in patients receiving HD, presepsin values were markedly high, at comparable levels to those of severe sepsis or septic shock [8,10]. In patients with chronic kidney disease (CKD) not receiving HD, presepsin levels inversely correlated with the measured GFR. These results suggest that the evaluation of presepsin levels in these patients requires special consideration.

We found that the presepsin values of HD patients without infection were 783–2,360 pg/ml. Liu et al. [14] reported that median presepsin values of in the ED were 787 pg/ml for severe sepsis and 1,084 pg/ml for septic shock. Recently, Nakamura et al. [18] analyzed presepsin levels in patients in the ICU with a loss of kidney function or end-stage renal disease according to RIFLE criteria. Presepsin levels in patients with sepsis ranged from 2,632 to 20,000 pg/ml and those in patients without sepsis ranged from 2,134 to 19,633 pg/ml, suggesting that presepsin measurement could not aid in distinguishing patients with sepsis from those with severe acute kidney injury [18].

However, the reason for its excessive elevation in patients receiving HD is unclear. We measured plasma presepsin concentration before and after the HD session, and found that presepsin levels significantly decreased after the session. The result implies that presepsin could be diffused and filtrated to a certain degree by high-flux HD. However, the values after HD were still high. We presume that elevated presepsin levels in patients receiving HD are due to decreased clearance and/or increased production of presepsin. Further study is needed to clarify this point.

In this study, presepsin levels inversely correlated with GFR (r = -0.68) in patients not receiving HD. The maximal presepsin value was 348 pg/ml (the measured GFR and C-reactive protein level of patients were 21.8 ml/min/1.73m^2^ and 0.06 mg/dl, respectively), and this values is under the cutoff value for diagnosing sepsis, which was reported to be approximately 400–600 pg/ml. However, this does not imply that the cutoff value is useful in all patients who are not receiving HD, because presepsin levels in patients with CKD who contract even a mild infection could easily surpass the value. Further study is needed to clarify the cutoff value for patients with CKD.

There were two strengths in this study. First, the GFR was measured using inulin renal clearance measurements in each patient. In past reports, the evaluation of GFR was based on eGFR calculated with age, gender, and serum creatinine concentration. However, the calculation of eGFR can be inaccurate because of underlying errors in the formula. Moreover, GFR could fluctuate in patients with sepsis because of potentially coexisting acute kidney injury [28]. Second, this study evaluated the independent effect of kidney function, because patients with infection, cancer, liver disease, autoimmune disorder, and steroid or immunosuppressant use, which could influence the presepsin levels, were carefully excluded.

We then sought to explore variables that correlated with presepsin values. Univariate analysis revealed apart from GFR, levels of serum albumin, serum uric acid, serum urea nitrogen, serum creatinine, and hemoglobin significantly correlated with presepsin levels. All these variables can intrinsically be associated with kidney function. Multivariate linear regression analysis revealed that the measured GFR and hemoglobin levels were independent predictors of presepsin level. It was reported that serum concentrations of inflammatory cytokines, such as interleukin-6 (IL-6) or tumor necrosis factor alpha (TNF-α), were elevated in patients with CKD [29,30,31]. IL-6 affect erythropoiesis through regulation of iron metabolism [32], and TNF-α inhibit erythroid cell development [33,34]. Recently, CKD patients reported to have high levels of circulating endotoxin compared to subjects with normal kidney function because of interstitial bacterial load or gut edema [35]. We supposed that this condition could lead to elevated circulating inflammatory cytokines, anemia and elevated production of presepsin, but further study is needed to elucidate this issue.

This study has limitations. First, this study was preliminary and we did not conduct legitimate sample size estimation. However, we calculated that this study has more than 80% power to detect a between-group difference of 0.8 SD, which corresponds to a large size difference in Cohen's criteria [36]. Second, the cutoff value for diagnosing sepsis in patients with a decreased GFR or in those receiving HD remains uncertain. Further study recruiting a larger number of patients with decreased GFR, including those with infection will be needed.

In conclusion, presepsin levels in patients receiving HD were markedly high, and were comparable to the levels seen in patients with severe sepsis or septic shock. In patients not receiving HD, presepsin levels increased as GFR decreased. Thus, the evaluation of presepsin in patients with CKD warrants special consideration, and a different cutoff value is needed for diagnosing sepsis in such patients.

## Supporting Information

S1 Table The anonymous data set of 71 patients. (CSV)
